# Changes in health-related quality of life and sleep habits after a 6-month non-randomised cluster-controlled trial among children with overweight or obesity

**DOI:** 10.1007/s00787-024-02375-0

**Published:** 2024-02-23

**Authors:** Annette Løvheim Kleppang, Eirik Abildsnes, Kristin Haraldstad, Tonje Holte Stea

**Affiliations:** 1 Faculty of Health and Sport Sciences, Department of Health and Nursing Science, University of Agder, Post-Box 422, 4604 Kristiansand, Norway; 2 Department of Public Health and Sport Sciences, Faculty of Social and Health Sciences, Inland Norway University of Applied Sciences, Elverum, Norway; 3 Institute of Health and Society, Faculty of Medicine, University of Oslo, Oslo, Norway

**Keywords:** Overweight, Obesity, Children, Family-based intervention, Sleep, Quality of life

## Abstract

Being overweight or obese can have severe negative psychological impacts and reduce health-related functioning. To improve health-related quality of life (HRQoL) and sleep habits for children with overweight or obesity, it is important to design and implement effective interventions. The aim of this study was to evaluate the effects of a 6-month family-based lifestyle intervention on HRQoL and sleep habits in Norwegian children with overweight or obesity in a primary-care setting. This 6-month, non-randomised, cluster-controlled trial included Norwegian children aged 5–13 years with overweight or obesity and their parents. A questionnaire was filled out by the parents. A total of 33 and 52 children in the control group and 41 and 78 children in the intervention group answered the HRQoL and sleep habits questions, respectively, and were included. The intervention group received individual family counselling and participated in physical activity groups and nutrition courses. The Children’s Sleep Habits Questionnaire (CSHQ) and Kidscreen-10 index were used to assess sleep habits and HRQoL. At baseline, the mean average scores for HRQoL were 50.0 [standard deviation (SD) 8.1] for the intervention group and 49.0 (SD 10.1) for the control group. For sleep habits at baseline, the mean average scores were 45.2 (SD 11.8) for the intervention group and 46.0 (SD 11.9) for the control group. No significant changes in HRQoL and sleep habits after the intervention were revealed. Overall, the family-based lifestyle intervention targeting overweight and obese children in a primary-care setting showed no significant effect on HRQoL or sleep quality.

## Introduction

Obesity is a major public-health challenge affecting children of all ages worldwide [1, 2]. In the World Health Organization (WHO) European region, 7.9% of children under 5 years of age and one in three school-aged children live with overweight or obesity [3]. In Norway, a longitudinal study conducted among children and adolescents aged 8–13 showed that the prevalence of overweight was 16% and the prevalence of obesity among adolescents was 3%, as well as that overweight and obesity in adolescence seemed to be established in childhood [4]. Obesity in childhood and adolescence is a complex condition caused by genetic, biological, cultural and environmental factors [5]. The childhood obesity epidemic is attributed to factors such as exposure to an obesogenic environment, sedentary behaviours, low levels of physical activity and unhealthy dietary habits. [6, 7]. Moreover, factors like gender, age, and familial predisposition to weight gain interact with each of these elements to determine the extent to which they act as protective or risk factors [7].

Being overweight or obese can have significant negative psychological impacts and result in the significant worsening of health-related functioning [8]. The results of previous studies confirm that childhood overweight and obesity often track into adulthood and may lead to an increased risk of chronic disorders [9, 10]. Furthermore, children and adolescents with obesity can develop a number of serious health-related problems, such as cardiovascular disease and diabetes [9, 11], insomnia [12, 13], mental health problems [14, 15], stigmatisation and reduced self-esteem and quality of life (QoL) [16, 17].

Poor sleep quality is associated with overweight and obesity, and some studies indicate that this relationship is independent of sleep duration [18]. Moreover, previous meta-analyses have reported short sleep duration to be a risk factor for or marker of the development of obesity in children and adolescents [18, 19], as well as that interventions to improve sleep duration may lead to reduced weight gain in preschool-aged children [19]. A recent systematic review that included 46 observational studies from 21 countries reported later sleep timing to be associated with poorer emotional regulation and cognitive function, lower physical activity, higher levels of sedentary behaviours, shorter sleep duration and unhealthy eating behaviours, but the evidence regarding the association between sleep timing and obesity was inconsistent; half of the included studies reported insignificant findings [20]. Obesity has also been identified as an independent risk factor for obstructive sleep apnoea in children and adolescents [21]. Previous studies have claimed that children with overweight have lower scores in the physical and self-esteem domains [22, 23]. Furthermore, childhood overweight and obesity are associated with an increased risk of experiencing mental disorders in childhood; adolescence and, potentially, adulthood [15]. Thus, prevalence of obesity and mental-health problems are closely related, and a bidirectional relationship has been suggested [24].

In epidemiology, health-related quality of life (HRQoL) has commonly been used to describe subjective perceptions of health and well-being [25]. A recent systematic review of studies examining QoL and obesity in paediatric population groups provides evidence for the impact of obesity on self-esteem and QoL [26]. Another literature review conducted among children with overweight has shown that the risk of impaired QoL is greater in clinical populations than the general population [27]. A previous study based on results from ten European countries has confirmed that children and adolescents with overweight and obesity have significantly lower HRQoL than their normal-weight peers [28]. Moreover, statistically significant improvements in HRQoL have been shown among children and adolescents with overweight or obesity after lifestyle interventions [29–31].

Socioeconomic status (SES) exerts a significant influence on the prevalence and effect of multiple risk factors for the development of childhood obesity and the persistence of overweight and obesity into adulthood [32]. Moreover, socioeconomic adversity has been highlighted as a very important risk factor during infancy and early childhood [33].

Diverse approaches targeting various populations have been employed to develop behavioural and lifestyle interventions to address childhood overweight and obesity. Interventions involving families typically encompass resources and assistance aimed at educating parents and children about nutrition, promoting physical activity and facilitating behavioural changes [34]. A systematic review showed that parental participation seems to play a constructive role in interventions aimed at preventing and treating overweight and obesity among children aged 3–12 years, focussing on nutrition and physical activity [35]. However, few studies have included parents as behavioural agents to improve sleep habits and QoL in children and adolescents with overweight or obesity. Early adolescence is a critical stage of development when lifestyle habits develop. Establishing healthy habits early in life can significantly reduce the risk of health problems in adulthood and have long-term health benefits [36]. The results of a systematic and evidence-based pilot study developed to specifically target children with overweight or obesity and their families have been published [37]. Based on the findings of this pilot study, a family-based, multi-component program with components tailored to the local context in Norwegian municipalities was developed to improve lifestyle behaviour among children and adolescents in a primary-care setting.

In conclusion, it is difficult to draw any firm conclusion based on the current knowledge base. Few previous studies have included parents as behavioural agents in intervention studies examining HRQoL and sleep habits among children and adolescents with overweight or obesity. This paper sets out to address this gap by evaluating the effects of a 6-month family-based lifestyle intervention on HRQoL and sleep habits in Norwegian children aged 5–13 years with overweight or obesity in municipal settings.

## Methods

### Study design and participants

This 6-month non-randomised cluster-controlled trial was developed and implemented as a tailored family-based intervention to improve lifestyle behaviours among children by encouraging healthy dietary habits and physical activity, as well as enhancing family engagement, parental self-efficacy and parent–child relationships [37].

The study population consisted of children with overweight or obesity who were recruited from October 2014 to September 2016 after routine height and weight measurements among preschoolers and third-grade pupils were taken by public health nurses. The International Obesity Task Force (IOTF) BMI cut-offs and IOTF LMS parameters were used to categorise BMI scores and calculate BMI z-scores, respectively [38]. Public health clinics (PHCs) and healthy life centres (HLCs) in nine municipalities were responsible for recruiting families and conducting the intervention in collaboration with local sports clubs. Some PHCs and HLCs facilitated the implementation of the intervention program and enlisted participants for the intervention group, while other PHCs and HLCs enlisted participants for the control group. The control group functioned as a waiting-list group and did not receive any guidance during the intervention period. Thus, the participants in the control group were informed that after serving as a control group for 6 months, they would receive the intervention program via PHCs and HLCs in their local communities. More details on the recruitment process and weighting have been previously published elsewhere [37].

The intervention and control groups were selected without any random pre-selection processes. A total of 166 children and adolescents aged 5–13 years and their families agreed to participate in the study (intervention group: *n* = 89, control group: *n* = 77). Causes of attrition included conflicting time schedules, a lack of time, participation in other leisure-time activities and relocation to a different geographical area. A total of 33 and 52 children in the control group and 41 and 78 children in the intervention group answered both the HRQoL and sleep habits questions at both baseline and 6-month follow-up, respectively.

**Fig. 1:**
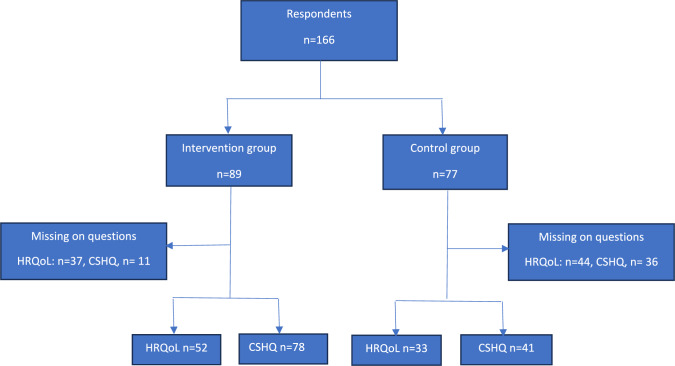
Flowchart of the study population at baseline and follow-up

Parents received written and oral information about the study. Those parents who agreed to participate signed a written consent form before completing an online questionnaire. The questionnaire provided information about their children’s lifestyle behaviours; demographic data and socio-demographic data, including educational attainment, age and sex (Fig. 1).

### Intervention

The intervention focussed on changing key behaviours (i.e., diet, level of physical activity and self-regulation skills) [37]. Tailored individual (face-to-face) counselling was received by all the parents in the intervention group, which was followed up on by certified health personnel using motivational interviewing (MI) to promote empowerment, internal motivation and the mastery of health [37]. A minimum of three and a maximum of eight individual consultations were offered to support all participating families during the intervention period. During these meetings between parents and health personnel, an individual plan was created defining a maximum of three main goals for behavioural change, followed by several more specific sub-goals for each family during the intervention period. To support all participating families in reaching these goals during the intervention period, three to eight individual consultations were offered.

The participants in the intervention group and their parents also attended a minimum of four and a maximum of five face-to-face half-day courses (4 h) during the intervention period, including both theoretical and practical learnings sessions intended to improve their dietary habits. The courses were developed by nutritionists and customised to meet the specific challenges and needs of the participants by improving nutritional knowledge, skills, attitudes and self-efficacy and, thus, improving dietary habits. Both group-based courses and individual counselling sessions focussed on changing the home environment by providing positive reinforcement, mobilising social support and focussing on the importance of the low availability of unhealthy foods and beverages and the high availability of healthy food options.

Additionally, children in the intervention group participated in physical activity (PA) groups for 2 h one to two times per week. The main aim of the PA sessions was to increase moderate-to-vigorous-intensity physical activity (MVPA) and improve motor control skills among the participants. The PA program was developed by sport scientists, who were experienced in planning, implementing and evaluating the effects of PA sessions targeting children with overweight and obesity. Detailed information about the program components and materials is provided elsewhere [37]. The health personnel responsible for the individual consultations and group-based courses, as well as the certified activity leaders responsible for the PA groups, ensured that the intervention’s adherence and fidelity were according to protocol.

### Questionnaire

Information about socio-demographic background, sleep patterns and QoL was provided by parents who completed online questionnaires using Survey Exact®. Weight and height were measured at baseline and after the 6-month intervention period by trained health professionals at the participating PHCs and HLCs. Each participant was given a personal identification number, which was used to identify and link their data.

#### HRQoL, Kidscreen-10 Index

Health-related quality of life in children was examined using the Kidscreen-10 index, which was developed from the longer Kidscreen-52 [25]. The Kidscreen-10 is a unidimensional instrument focussing on the functional, mental and social aspects of well-being in children and adolescents 8–18 years of age. In the present sample, a proxy version was administered to the parents. The instrument consists of several items, beginning with ‘Thinking of last week, has your child… (1) felt fit and well, (2) felt full of energy, (3) felt sad, (4), felt lonely, (5) had enough time for him/herself, 6) been able to do the things that he/she want in his/her free time, (7) felt that his/her parent(s) treated him/her fairly, (8) had fun with his/her friends, (9) got on well at school and (10) been able to pay attention at school?’ There are five response categories for each item, ranging from ‘not at all’ to ‘extremely’ or from ‘never’ to ‘always’, indicating either the intensity of an attitude or the frequency of a behaviour or feeling. The total score is calculated based on item-response theory and transformed to yield scores with a mean of 50 and a standard deviation of 10 [39]. The Kidscreen-10 has been demonstrated to be a valid measure of general HRQoL factors [40], and a cutoff value of > 0.41 has been developed [41]. The instrument has also been used in previous studies examining HRQoL in overweight and obese children and adolescents [42, 43].

#### Sleep habits

Sleep habits were measured with the Children’s Sleep Habits Questionnaire (CSHQ). The CSHQ was designed to reflect common clinical symptoms presenting in school-aged children (4–10 years old) [44]. The instrument is a retrospective, 33-item scored parent questionnaire that includes items relating to bedtime behaviour and sleep onset, sleep duration, anxiety around sleep, behaviour during sleep and night wakings, sleep-disordered breathing, parasomnias and morning waking/daytime sleepiness [44]. Parents reported poor sleep behaviour on the part of their child during a typical week as follows: ‘Usually’ (5–7 times/week), ‘Sometimes’ (2–4 times/week) or ‘Rarely’ (0–1 times/week). Higher scores indicated worse sleep behaviours or problems. The CSHQ is a multi-dimensional tool that is widely used to screen for paediatric sleep problems [45]. Some items were reversed to consistently make a higher score indicative of more disturbed sleep. A Total Sleep Disturbances score, which can range from 33 to 99, is calculated as the sum of all scored CSHQ questions, and a score above 41 indicates a paediatric sleep disorder [44]. The CSHQ has demonstrated adequate psychometric properties and been shown to be a useful instrument for clinical and research settings [46].

#### Parental education, child’s sex, age, BMI and BMI z-scores

The questionnaire also included questions about parental education, child’s sex and child’s age. Parental education level was assessed with the following question: *What level of education do you have; answer for yourself and your partner.* Both questions (for yourself and your partner) had six response options: *elementary school* < *7 years; elementary school 7–10 years; vocational school/high school* < *3 years; high school* > *3 years; high school, college, or university* ≤ *4 years* and *colleges or university* ≥ *4 years.* These response alternatives were then trichotomised to reflect the following educational levels for both parents: primary school, high school and college/university. For each participant, BMI was calculated (body mass (kg)/height^2^ (m^2^), and a BMI z-score was assigned [47].

### Statistical analysis

Differences in HRQoL, sleep patterns and socio-demographic characteristics between the intervention and control groups at baseline were analysed using an independent-sample *t *test for continuous variables and a Chi-square test for categorical variables. Differences in HRQoL and sleep patterns between the intervention group and the control group at baseline and after the intervention period were compared using a Chi-square test. The effect of the intervention on HRQoL and sleep patterns was tested using multiple multi-level linear regression models. All models were adjusted for baseline scores, age, sex, maternal and paternal education and BMI *z*-scores. Statistical analyses were performed using the Statistical Package for Social Sciences (SPSS), version 26 (IBM Corporation). For all analyses, *p* values < 0.05 were considered statistically significant.

## Results

Table 1 shows the baseline characteristics of the intervention and control groups.

**Table 1:** Baseline characteristics of the intervention and control group

	Intervention group (*n* = 89)	Control group (*n* = 77)	*p* value*
Age, mean (SD)	8.5 (2.1)	8.1 (1.2)	0.114
Gender, *n* (%)			
Female	54 (60.7)	45 (58.4)	0.770
Male	35 (39.3)	32 (41.6)	
Maternal educational level, *n* (%)			
Primary school	6 (8.7)	3 (5.2)	0.286
High school	31 (44.9)	34 (58.6)	
Colleague/ University	32 (46.4)	21 (36.2)	
Paternal educational level			
Primary school	8 (11.6)	9 (16.1)	0.069
High school	33 (47.8)	35 (62.5)	
Colleague/ University	28 (40.6)	12 (21.4)	
BMI *z*-score, mean (SD)	2.0 (0.4)	2.0 (0.4)	0.827
IsoBMI, *n* (%)			
≥ 25 overweight	14 (34.1)	12 (36.3)	0.382
≥ 30 obesity	14 (34.1)	15 (45.5)	
≥ 35 severe obesity	13 (31.7)	6 (18.2)	

^*^Tested using independent sample *t* test for continuous variables (age, BMI) and Chi-square test for categorical variables

Baseline characteristics revealed no differences in the prevalence of overweight, obesity, age, sex, maternal and paternal education between the intervention group and the control group at baseline (Table 1).

Table 2 presents the effects of the intervention on HRQoL and sleep habits at follow-up.

**Table 2:** Changes in health-related quality of life and sleep habits

	Baseline			6 months of follow-up			
	Intervention group	Control group	*p* value^a^	Intervention group	Control group	*p* value^a^	β (95% CI)^b^
Health-related quality of life (HRQL)							
Mean (SD)	50.0 (8.1)	49.0 (10.1)	0.642	50.6 (8.8)	47.8 (7.9)	0.152	0.20 (− 0.41, 6.23)
HRQoL (*n*, %)							
Less HRQoL	5 (12.2)	10 (30.3)	0.054*	7 (17.1)	6 (18.2)	0.901	− 0.12 (− 0.20, 0.18)
Better HRQoL	36 (87.8)	23 (69.7)		34 (82.9)	27 (81.8)		
Sleep habits (CSHQ)							
Mean (SD), total	45.2 (11.8)	46.0 (11.9)	0.692	42.5 (13.7)	45.7 (10.1)	0.129	− 0.02 (− 7.94, 6.76)
Subscales (CSHQ)							
Bedtime resistance	10.2 (2.4)	11.1 (2.2)	0.045*	10.1 (2.6)	10.9 (1.9)	0.077	− 0.07 (− 2.22, 1.35)
Sleep onset delay	2.6 (0.6)	2.4 (0.7)	0.121	2.6 (0.6)	2.3 (0.6)	0.021**	0.30 (− 0.03, 0.71)
Sleep duration	6.7 (1.0)	6.5 (1.1)	0.283	6.4 (1.6)	6.6 (0.9)	0.285	− 0.05 (− 1.16, 0.83)
Sleep anxiety	5.5 (1.7)	5.9 (2.4)	0.268	5.5 (2.1)	6.1 (2.0)	0.120	− 0.15 (− 1.82, 0.63)
Night waking	3.6 (1.3)	3.6 (1.3)	0.998	3.3 (1.5)	3.5 (1.3)	0.528	− 0.09 (− 1.26, 0.68)
Parasomnia	7.9 (3.0)	7.8 (2.8)	0.767	7.5 (3.5)	7.4 (2.4)	0.813	− 0.08 (− 2.33, 1.21)
Sleep-disordered breathing	3.5 (1.2)	3.3 (1.2)	0.474	3.1 (1.6)	3.4 (1.4)	0.406	0.01 (− 1.14, 1.23)
Daytime sleepiness	10.0 (3.4)	10.2 (3.4)	0.731	9.8 (3.4)	10.4 (3.0)	0.367	0.03 (− 1.83, 2.41)
Insomnia (*n*, %)							
Low sleep problems	17 (21.8)	13 (25.0)	0.671	28 (35.9)	10 (19.2)	0.041	− 0.06 (− 0.34, 0.21)
Sleep problems	61 (78.2)	39 (75.0)		50 (64.1)	42 (80.8)		

A total of 33 and 52 children in the control group and 41 and 78 children in the intervention group answered the HRQoL and sleep habits questions and were included

*HRQoL* health-related quality of life (proxy version), *SD* standard deviation. KIDSCREEN-10. Rasch scores were computed and transformed into *t* values, with a mean of 50 and an SD of 10. Higher values indicate higher levels of HRQoL

*CSHQ* Child Sleep habits Questionnaire, higher values indicate more sleep problems

^a^Tested using independent sample t test for continuous variables (HRQoL and sleep habits), and Chi-square tests for insomnia and HRQoL with a cut off value off > 41 for both insomnia and HRQoL. These tests are used to examine differences between the intervention group and the control group both at baseline and 6 months follow-up

^b^Intervention effect analyzed as difference in mean scores at baseline and after 6 months of follow-up in each variable (of each group) adjusted for baseline scores, age, sex, gender, BMI *z*-score, paternal-and maternal educational level using linear regression models

At baseline (Table 2), the results showed an average HRQoL score of 50.0 [standard deviation (SD) 8.1] for the intervention group and 49.0 (SD 10.1) for the control group. For sleep habits, the average score for the intervention group was 45.2 (SD 11.8) and that for the control group was 46.0 (11.9). The results indicated no significant changes in HRQoL and sleep habits because of the intervention. Significant differences in HRQoL, using the cutoff value to identify those with worse and better HRQoL, were observed at baseline between the control and intervention groups. Furthermore, significant differences in insomnia levels at baseline for the bedtime-resistance subgroup. At 6-month follow-up, for the sleep subgroup, onset delay was observed between the control and intervention groups. A decrease in insomnia for the intervention group (78.2% versus 64.1%) and an increase in insomnia for the control group (75.0% versus 80.8%) were observed, but no significant intervention effect was found.

## Discussion

The present study assessed the effects of a complex intervention program on HRQoL and sleep patterns in children with overweight or obesity. Overall, we found no effect on the part of the intervention on proxy-reported HRQoL or sleep habits after 6 months. Our findings are in line with a school-based PA intervention targeting the general population, which demonstrated little or no effect on HRQoL [48]. However, that study used the Kidscreen-27, measured PA objectively, was cluster-randomised by school and included children and adolescents with a higher mean age than our study. A school-based intervention program intended to promote healthy lifestyles in children with obesity aged 10–12 reported improved HRQoL after a 6-month program period [49]. Improvement in participants’ HRQoL was also found in a study of overweight and obese children aged 7–13 after the completion of a 10-week lifestyle-intervention program [31]. Whereas our study used the Kidscreen-10 index, the two latter studies used the Paediatric Quality of Life Inventory to measure HRQoL. A study conducted among children aged 8–19 with severe obesity found significant improvements in HRQoL after a 1-year lifestyle intervention, and higher weight loss was associated with higher HRQoL, as measured with the Kidscreen-52 [29]. However, the study included children and adolescents with a higher mean age than those our study, the study had a longer intervention period and only children with severe obesity were included. How sensitive the Kidscreen-10 index is when measuring change related to dietary habits and PA in studies of children with overweight or obesity should be examined in future studies. However, studies have demonstrated the instrument’s ability to detect changes over time [50]. The sensitivity of the Kidscreen-27 has been investigated in a previous cluster-randomised controlled trial [48].

It should be noted that the average scores reflecting baseline HRQoL in the present study were relatively high. In line with our results, another intervention study targeting overweight and obese children presented high mean scores for HRQoL at baseline, which may partially explain the lack of an intervention effect on HRQoL [31]. Moreover, the baseline results reflecting HRQoL in the present study are in line with other previous studies and normative data [25, 28, 51]. Similar results may be partially explained by the low mean age of the participants in the present study because all were under the age of 13. Proxy measures were used in this study, and parent–child agreement on the Kidscreen has been mixed [43]. In general, there is moderate correspondence between the child’s self-report and the parent’s proxy report in non-clinical populations [52, 53]. A follow-up study from Spain showed low to moderate agreement between children aged 8–18 and parental proxies during both baseline and follow-up assessments, and agreement was lower in the follow-up study [54]. Parents and children may differ in their understandings and interpretations of the items. However, proxy reports are valuable because the parents’ perceptions of their children’s HRQoL may contribute to their own actions and reactions [53].

Furthermore, we did not observe a significant effect on the part of the intervention on sleep habits, which is in line with a recent randomised intervention study on psychological stress and sleep habits among obesity-susceptible healthy-weight children and their parents [55]. However, the present study showed a significant difference in sleep-onset-delay at the 6-month follow-up between the control and intervention groups. The present study also found that 78.2% of children in the intervention group and 75.0% of children in the control group had insomnia at baseline. This finding corresponds with earlier research indicating a high prevalence of mild to moderate sleep-related breathing issues in children with obesity [56], as well as a relationship between shorter sleep duration during middle childhood and an elevated risk of obesity in early adulthood [57]. There is also some evidence suggesting that short sleep duration is linked to obesity through low PA levels and poor diet [58]. Additionally, relationships have been shown between insufficient sleep, poor dietary habits and obesity [59]. A previous meta-analysis of prospective studies involving children and adolescents concluded that short sleep duration was a risk factor for the development of obesity [18]. Furthermore, a meta-review provided consistent evidence linking short sleep duration to a high prevalence of adiposity and adverse emotional outcomes [60]. However, the association between poor sleep and the risk of developing metabolic syndrome in children appeared to be weak and inconsistent in the meta-review. The lack of a significant intervention effect on sleep habits in the present study may be attributable to insufficient power due to the small sample size, leading to a potential lack of power to demonstrate a difference. Another possible explanation may be that the children in our sample had scores for sleep habits (mean values of 45.2 and 46.0) that are higher than those reported among children in the general population in other countries, such as 40.7 for the Netherlands, 38.7 for the US, 42.1 for China and 40.7 for Germany [61]. However, when comparing our results to those of studies on sleep habits among younger children, we find that they were generally consistent or that the scores in the latter studies were slightly higher. For example, Italian preschool children aged 3–6 years had a mean value of 47.0 [62], and Brazilian children aged 4–10 years had a mean value of 46.9 [63].

Furthermore, common background factors, such as SES, may influence both HRQoL and various lifestyle behaviours. The results highlight the need for a broader approach in family-based intervention programs, including PA, diet and concerns regarding SES, to promote HRQoL and sleep habits, specifically in relation to overweight and obese children. Both HRQoL and sleep habits are complex phenomena, and the way these concepts are defined and measured, along with considerations of PA and diet, can have a significant impact on children with overweight or obesity. Moreover, there may be a lag effect before a lifestyle intervention leads to changes in sleep habits and HRQoL. For example, it may take time until new key behaviours (i.e., diet, level of PA and self-regulation skills) are mastered by parents, children, and adolescents. Thus, one interesting issue is how much change in PA and diet is needed to influence sleep habits and HRQoL in relatively healthy children and adolescents. One reason for no significant effect on HRQoL or sleep quality could be that key behaviours (exposures) in the intervention groups have generally been insufficient.

### Strength and limitations

A strength of the present study was the use of a structural theory and evidence framework for the development, implementation and evaluation of the intervention. Additionally, the results of the pilot study provided information on barriers and opportunities, allowing us to tailor the intervention to the local context in Norwegian municipalities [37]. The use of pilot study also ensured the intervention materials and tools were tested and adapted to the health personnel conducting the study, as well as the target group. Another strength was the use of validated measures (HRQoL and the CSHQ) and the use of height and weight values that were objectively measured by trained public health nurses. Additionally, the study was conducted in ordinary municipal services, making it possible to implement similar activities in other locations when assessing longitudinal associations.

Furthermore, the effect analyses for the intervention were adjusted for well-known confounders, such as sex, age, BMI z-score and paternal and maternal educational levels. However, we cannot exclude potential residual confounders attributable to unknown or unmeasured factors; for example, when assessing the intervention effect of diet and PA, diet and PA may also be confounders. In addition, the current controlled trial lacks a randomised design, and it has a short follow-up and a small sample size. Moreover, as municipal healthcare institutions have a responsibility to prevent and treat overweight and obesity among all age groups [64], we used a waiting-list design, which did not allow for long-term controlled follow-up. Another limitation was the fact that despite mandatory weight and height screening among preschoolers and third graders, in several municipalities, the recruitment process in regular services did not allow us to control this process and ensure that all children and families in the target group had been invited to participate. Moreover, high drop-out rates resulted in low numbers of participants in the control and intervention groups and made it impossible to analyse long-term data (12 + 24 months), as was originally planned. The attrition rate for the HRQoL measurements was high, and the HRQoL results must be interpreted with caution. Furthermore, the lack of change in HRQoL during the intervention period may be attributable to insufficient power due to the narrow range of BMI scores and small sample size [65].

## Conclusions

Overall, the family-based lifestyle intervention targeting overweight and obese children showed no significant effect on HRQoL or sleep quality. Both HRQoL and sleep habits are complex phenomena. Therefore, future research with larger sample sizes and tailored interventions is crucial to gaining a deeper understanding of how PA and diet influence HRQoL and sleep patterns in this population.

## Data Availability

The data are available from the corresponding author on reasonable request.

## References

[1] Gurnan M, Birken C, Hamilton J (2015) Childhood obesity: causes, consequences, and management. Pediatr Clin N Am 62(4):821–840 10.1016/j.pcl.2015.04.001 26210619

[2] Sahoo K et al (2015) Childhood obesity: causes and consequences. J Family Med Prim Care 4(2):187–192 25949965 10.4103/2249-4863.154628 PMC4408699

[3] World Health Organization (2022) WHO European regional obesity report. https://www.who.int/europe/publications/i/item/9789289057738. Accessed 21 Dec 2023

[4] Øvrebø B et al (2021) Overweight, obesity, and thinness among a nationally representative sample of Norwegian adolescents and changes from childhood: associations with sex, region, and population density. PLoS ONE 16(8):e0255699 34343207 10.1371/journal.pone.0255699 PMC8330951

[5] Kansra AR, Lakkunarajah S, Jay MS (2021) Childhood and adolescent obesity: a review. Front Pediatr 8:581461 33511092 10.3389/fped.2020.581461 PMC7835259

[6] Kaczynski AT et al (2020) Development of a national childhood obesogenic environment index in the United States: differences by region and rurality. Int J Behav Nutr Phys Act 17(1):83 32615998 10.1186/s12966-020-00984-x PMC7330993

[7] Davison KK, Birch LL (2001) Childhood overweight: a contextual model and recommendations for future research. Obes Rev 2(3):159–171 12120101 10.1046/j.1467-789x.2001.00036.x PMC2530932

[8] Flodmark CE (2005) The happy obese child. Int J Obes 29(2):S31–S33 10.1038/sj.ijo.0803060 16385749

[9] Llewellyn A et al (2016) Childhood obesity as a predictor of morbidity in adulthood: a systematic review and meta-analysis. Obes rev 17(1):56–67 26440472 10.1111/obr.12316

[10] Simmonds M et al (2016) Predicting adult obesity from childhood obesity: a systematic review and meta-analysis. Obes rev 17(2):95–107 26696565 10.1111/obr.12334

[11] Friedemann C et al (2012) Cardiovascular disease risk in healthy children and its association with body mass index: systematic review and meta-analysis. BMJ 345:e4759 23015032 10.1136/bmj.e4759 PMC3458230

[12] Cai G-H et al (2018) Both weight at age 20 and weight gain have an impact on sleep disturbances later in life: Results of the EpiHealth study. Sleep 41(1):zsx176 10.1093/sleep/zsx176 29361188

[13] Lin C-Y et al (2020) Psychological distress and quality of life in Iranian adolescents with overweight/obesity: Mediating roles of weight bias internalization and insomnia. Eat Weight Disord-St 25(6):1583–1592 10.1007/s40519-019-00795-5 31673986

[14] Godina-Flores NL et al (2022) Obesity and its association with mental health among Mexican children and adolescents: systematic review. Nutr Rev 81:658–669 10.1093/nutrit/nuac083 PMC10170326 36164834

[15] Rankin J et al (2016) Psychological consequences of childhood obesity: psychiatric comorbidity and prevention. Adolesc Health Med Ther 7:125–146 27881930 10.2147/AHMT.S101631 PMC5115694

[16] Alimoradi Z et al (2020) Weight-related stigma and psychological distress: a systematic review and meta-analysis. Clin Nutr 39(7):2001–2013 31732288 10.1016/j.clnu.2019.10.016

[17] Lindberg L et al (2020) Anxiety and depression in children and adolescents with obesity: a nationwide study in Sweden. BMC med 18(1):1–9 32079538 10.1186/s12916-020-1498-z PMC7033939

[18] Miller MA et al (2018) Sleep duration and incidence of obesity in infants, children, and adolescents: a systematic review and meta-analysis of prospective studies. Sleep 41(4):zsy018 10.1093/sleep/zsy018 29401314

[19] Miller MA et al (2021) Systematic review and meta-analyses of the relationship between short sleep and incidence of obesity and effectiveness of sleep interventions on weight gain in preschool children. Obes Rev 22(2):e13113 33237635 10.1111/obr.13113

[20] Dutil C et al (2022) Sleep timing and health indicators in children and adolescents: a systematic review. Health Promot Chronic Dis Prev Can 42(4):150–169 35481337 10.24095/hpcdp.42.4.04 PMC9116724

[21] Marcus CL et al (2012) Diagnosis and management of childhood obstructive sleep apnea syndrome. Pediatrics 130(3):e714–e755 22926176 10.1542/peds.2012-1672

[22] Lin YC (2019) The predictive relationship of health related quality of life on objectively-measured sleep in children: a comparison across BMI Ranges. Front Neurosci 13:1003 31611763 10.3389/fnins.2019.01003 PMC6775809

[23] van der Heijden LB et al (2021) Quality of life of children and adolescents with clinical obesity, perspectives of children and parents. Obes Res Clin Pract 15(5):466–472 34330695 10.1016/j.orcp.2021.07.001

[24] Reeves GM, Postolache TT, Snitker S (2008) Childhood obesity and depression: connection between these growing problems in growing children. Int J Child Health Hum Dev 1(2):103 18941545 PMC2568994

[25] Ravens-Sieberer U et al (2014) The European KIDSCREEN approach to measure quality of life and well-being in children: development, current application, and future advances. Qual Life Res 23(3):791–803 23686556 10.1007/s11136-013-0428-3 PMC3953538

[26] Griffiths LJ, Parsons TJ, Hill (2010) AJ Self-esteem and quality of life in obese children and adolescents: a systematic review. Int J Pediatr Obes 5(4):282–304 20210677 10.3109/17477160903473697

[27] Buttitta M et al (2014) Quality of life in overweight and obese children and adolescents: a literature review. Qual Life Res 23(4):1117–1139 24249217 10.1007/s11136-013-0568-5

[28] Ottova V et al (2012) Overweight and its impact on the health-related quality of life in children and adolescents: results from the European KIDSCREEN survey. Qual Life Res 21(1):59–69 21557001 10.1007/s11136-011-9922-7

[29] Hoedjes M et al (2018) Health-related quality of life in children and adolescents with severe obesity after intensive lifestyle treatment and at 1-year follow-up. Obes Facts 11(2):116–128 29631271 10.1159/000487328 PMC5981677

[30] Wong WW et al (2013) A residential summer camp can reduce body fat and improve health-related quality of life in obese children. J Pediatr Gastroenterol Nutr 56(1):83–85 22995865 10.1097/MPG.0b013e3182736f70

[31] van den Eynde E et al (2020) Changes in the health-related quality of life and weight status of children with overweight or obesity aged 7–13 years after participating in a 10-week lifestyle intervention. Child Obes 16(6):412–420 32706996 10.1089/chi.2020.0070 PMC7475087

[32] Vazquez CE, Cubbin C (2020) Socioeconomic status and childhood obesity: a review of literature from the past decade to inform intervention research. Curr Obes Rep 9(4):562–570 32785878 10.1007/s13679-020-00400-2

[33] Hemmingsson E (2018) Early childhood obesity risk factors: socioeconomic adversity, family dysfunction, offspring distress, and junk food self-medication. Curr Obes Rep 7(2):204–209 29704182 10.1007/s13679-018-0310-2 PMC5958160

[34] Brown HE, Schiff A, van Sluijs EMF (2015) Engaging families in physical activity research: a family-based focus group study. BMC Public Health 15(1):1178 26607429 10.1186/s12889-015-2497-4 PMC4660685

[35] Mead E et al (2017) Diet, physical activity and behavioural interventions for the treatment of overweight or obese children from the age of 6–11 years. Cochrane Database Syst Rev. 10.1002/14651858.CD012651 28639319 10.1002/14651858.CD012651 PMC6481885

[36] Shapiro MA, Nguyen ML (2010) Psychosocial stress and abdominal pain in adolescents. Ment Health Fam Med 7(2):65–69 22477924 PMC2939458

[37] Stea TH et al (2016) Using the intervention mapping protocol to develop a family-based intervention for improving lifestyle habits among overweight and obese children: study protocol for a quasi-experimental trial. BMC Public Health 16(1):1092 27756346 10.1186/s12889-016-3766-6 PMC5070224

[38] Cole TJ, Lobstein T (2012) Extended international (IOTF) body mass index cut-offs for thinness, overweight and obesity. Pediatr Obes 7(4):284–294 22715120 10.1111/j.2047-6310.2012.00064.x

[39] Europe T (2006) The KIDSCREEN Questionnaires. Quality of life questionnaires for children and adolescents. Pabst Science Publishers, Lengerich

[40] Ravens-Sieberer U et al (2010) Reliability, construct and criterion validity of the KIDSCREEN-10 score: a short measure for children and adolescents’ well-being and health-related quality of life. Qual life Res 19(10):1487–1500 20668950 10.1007/s11136-010-9706-5 PMC2977059

[41] Hirschfeld G, von Brachel R, Thiele C (2020) Screening for health-related quality of life in children and adolescents: optimal cut points for the KIDSCREEN-10 for epidemiological studies. Qual Life Res 29(2):529–536 31620984 10.1007/s11136-019-02324-4

[42] Evaristo S et al (2018) Associations between health-related quality of life and body mass index in Portuguese adolescents: LabMed physical activity study. Int J Adolesc Med Health 31(5):20170066 10.1515/ijamh-2017-0066 29432203

[43] Helseth S, Haraldstad K, Christophersen KA (2015) A cross-sectional study of Health Related Quality of Life and body mass index in a Norwegian school sample (8–18 years): a comparison of child and parent perspectives. Health Qual Life Outcomes 13:47 25884676 10.1186/s12955-015-0239-z PMC4396077

[44] Owens JA, Spirito A, McGuinn M (2000) The Children’s Sleep Habits Questionnaire (CSHQ): Psychometric properties of a survey instrument for school-aged children. Sleep 23(8):1043–1051 11145319

[45] Bonuck KA et al (2017) Modified children’s sleep habits questionnaire for behavioral sleep problems: a validation study. Sleep Health 3(3):136–141 28526249 10.1016/j.sleh.2017.03.009 PMC5473153

[46] de la Cruz L et al (2016) Reliability and validity of the Spanish version of the Children’s Sleep Habits Questionnaire (CSHQ-SP) in school-age children. Child Care Health Dev 42(5):675–682 27279384 10.1111/cch.12357

[47] Cole TJ, Freeman JV, Preece MA (1995) Body mass index reference curves for the UK, 1990. Arch Dis Child Educ Pract Ed 73(1):25–29 10.1136/adc.73.1.25 PMC1511150 7639544

[48] Resaland GK et al (2019) Effects of a physical activity intervention on schoolchildren’s health-related quality of life: the active smarter kids (ASK) cluster-randomized controlled trial. Prev Med Rep 13:1–4 30456052 10.1016/j.pmedr.2018.11.002 PMC6234767

[49] Morano M et al (2016) A multicomponent, school-initiated obesity intervention to promote healthy lifestyles in children. Nutrition 32(10):1075–1080 27209213 10.1016/j.nut.2016.03.007

[50] Palacio-Vieira JA et al (2008) Changes in health-related quality of life (HRQoL) in a population-based sample of children and adolescents after 3 years of follow-up. Qual Life Res 17(10):1207–1215 18931941 10.1007/s11136-008-9405-7

[51] Mikkelsen HT et al (2022) Changes in health-related quality of life in adolescents and the impact of gender and selected variables: a 2-year longitudinal study. Health Qual Life Outcomes 20(1):123 35982467 10.1186/s12955-022-02035-4 PMC9387404

[52] Eiser C, Morse R (2001) Can parents rate their child’s health-related quality of life? Results of a systematic review. Qual Life Res 10(4):347–357 11763247 10.1023/a:1012253723272

[53] Riiser K et al (2020) Confirmatory factor analysis of the proxy version of Kidscreen-27 and relationships between health-related quality of life dimensions and body mass index and physical activity in young schoolchildren. Prev Med Rep 20:101210 32995148 10.1016/j.pmedr.2020.101210 PMC7516181

[54] Rajmil L et al (2014) Socioeconomic inequalities in mental health and health-related quality of life (HRQOL) in children and adolescents from 11 European countries. Int J Public Health 59(1):95–105 23793782 10.1007/s00038-013-0479-9

[55] Olsen NJ et al (2022) Effects of the healthy start randomized intervention on psychological stress and sleep habits among obesity-susceptible healthy weight children and their parents. PLoS ONE 17(3):e0264514 35271601 10.1371/journal.pone.0264514 PMC8912262

[56] Danielsen YS et al (2022) Polysomnographic comparison of sleep in children with obesity and normal weight without suspected sleep-related breathing disorder. Clinical Obesity 12(1):e12493 34781415 10.1111/cob.12493

[57] Hart CN, Cairns A, Jelalian E (2011) Sleep and obesity in children and adolescents. Pediatr Clin N Am 58(3):715–733 10.1016/j.pcl.2011.03.007 PMC3107702 21600351

[58] Sluggett L, Wagner SL, Harris RL (2019) Sleep duration and obesity in children and adolescents. Can J Diabetes 43(2):146–152 30266216 10.1016/j.jcjd.2018.06.006

[59] Chaput JP (2014) Sleep patterns, diet quality and energy balance. Physiol Behav 134:86–91 24051052 10.1016/j.physbeh.2013.09.006

[60] Matricciani L et al (2019) Children’s sleep and health: a meta-review. Sleep med 46:136–150 10.1016/j.smrv.2019.04.011 31121414

[61] van Litsenburg RRL et al (2010) Sleep habits and sleep disturbances in Dutch children: a population-based study. Eur J Pediatr 169(8):1009–1015 20191392 10.1007/s00431-010-1169-8 PMC2890079

[62] Lionetti F et al (2021) The Children’s Sleep Habits Questionnaire: identification of sleep dimensions, normative values, and associations with behavioral problems in Italian preschoolers. Sleep Health 7(3):390–396 33867310 10.1016/j.sleh.2021.03.002

[63] Gios TS et al (2022) Translation and adaptation into Brazilian Portuguese and investigation of the psychometric properties of the Children’s Sleep Habits Questionnaire (CSHQ-BR). Sleep Med 100:550–557 36308913 10.1016/j.sleep.2022.10.001

[64] Health NDo Nasjonale faglige retningslinjer for primærhelsetjenesten. Forebygging og behandling av overvekt og fedme hos barn og unge. [National guideline for the prevention, identification and treatment of overweight and obesity in children and adolescents.]. 2010, Helsedirektoratet Oslo

[65] Stern M et al (2007) Gender, ethnicity, psychosocial factors, and quality of life among severely overweight, treatment-seeking adolescents. J Pediatr Psychol 32(1):90–94 16818482 10.1093/jpepsy/jsl013

